# Poly(I:C) Enhances the Susceptibility of Leukemic Cells to NK Cell Cytotoxicity and Phagocytosis by DC

**DOI:** 10.1371/journal.pone.0020952

**Published:** 2011-06-17

**Authors:** Eva Lion, Sébastien Anguille, Zwi N. Berneman, Evelien L. J. M. Smits, Viggo F. I. Van Tendeloo

**Affiliations:** 1 Vaccine & Infectious Disease Institute (Vaxinfectio), Laboratory of Experimental Hematology, Faculty of Medicine, University of Antwerp, Antwerp, Belgium; 2 Center for Cellular Therapy and Regenerative Medicine, Antwerp University Hospital, Antwerp, Belgium; Centre de Recherche Public de la Santé (CRP-Santé), Luxembourg

## Abstract

α Active specific immunotherapy aims at stimulating the host's immune system to recognize and eradicate malignant cells. The concomitant activation of dendritic cells (DC) and natural killer (NK) cells is an attractive modality for immune-based therapies. Inducing immunogenic cell death to facilitate tumor cell recognition and phagocytosis by neighbouring immune cells is of utmost importance for guiding the outcome of the immune response. We previously reported that acute myeloid leukemic (AML) cells in response to electroporation with the synthetic dsRNA analogue poly(I:C) exert improved immunogenicity, demonstrated by enhanced DC-activating and NK cell interferon-γ-inducing capacities. To further invigorate the potential of these immunogenic tumor cells, we explored their effect on the phagocytic and cytotoxic capacity of DC and NK cells, respectively. Using single-cell analysis, we assessed these functionalities in two- and three-party cocultures. Following poly(I:C) electroporation AML cells become highly susceptible to NK cell-mediated killing and phagocytosis by DC. Moreover, the enhanced killing and the improved uptake are strongly correlated. Interestingly, tumor cell killing, but not phagocytosis, is further enhanced in three-party cocultures provided that these tumor cells were upfront electroporated with poly(I:C). Altogether, poly(I:C)-electroporated AML cells potently activate DC and NK cell functions and stimulate NK-DC cross-talk in terms of tumor cell killing. These data strongly support the use of poly(I:C) as a cancer vaccine component, providing a way to overcome immune evasion by leukemic cells.

## Introduction

Active specific immunotherapy (ASI) for cancer aims at stimulating the host's immune system to recognize and eradicate tumor cells. Despite a sound conceptual basis and the observation of tangible clinical effects in some patients, cancer vaccination studies have so far shown only modest clinical benefit. To move the field forward, researchers are currently exploring new approaches to amend the therapeutic potential of ASI approaches. Over time it has become clear that combined therapies are likely to be the most successful in the fight against cancer. In this regard, there is strong interest in simultaneous targeting of multiple immune cells of the host's anti-tumor immunity on one hand and the immunogenic properties of the tumor cell compartment on the other hand [1]. In particular, concomitant activation of dendritic cells (DC) and natural killer (NK) cells and the promotion of NK-DC cross-talk is currently an attractive modality for ASI [2]-[4]. Dendritic cells are the key orchestrators of the immune system, bridging innate and adaptive immunity. Through uptake of dying cells, DC navigate the immune response to tolerance or immunity to antigens [5]. One of the challenges in the design of cancer vaccines is the promotion of the processing of exogenous tumor antigens by DC and the subsequent antigen presentation to specific CD8^+^ T cells (i.e. cross-presentation [6], [7]). In this context, exposure of DC to danger signals is crucial for their activation and the development of an efficient T helper type 1 (Th1)-mediated cellular immune response, desirable in cancer immune-based therapies [8]. Natural killer cells, as central players of the innate immune system, currently evoke a reinvigorated interest for their potential in ASI [9]–[11]. The direct anti-tumor effects of NK cells can be ascribed to their cytotoxic and cytokine-secreting capacity. Additionally, NK cells can also indirectly contribute to tumor control by communicating with DC and other immune cells, supporting the development of an efficient adaptive anti-tumor immune response [2], [12].

In light of cranking up the immunogenic properties of the tumor cells, the induction of immunogenic cell death has been shown to be of utmost importance in guiding the outcome of the triggered immune response [5], [13]. Tumor cell death can be induced in various ways, such as irradiation, hyperthermia, drug/chemical-induced, freeze-thawing, oncolytic virus infection, Toll-like receptor (TLR) signaling, or combinations of the above mentioned. For certain methods, dying immunogenic tumor cells were shown to be efficiently cross-presented via DC, exerting strong anti-tumor responses *in vitro* [14]–[24] and *in vivo* [23], [25]–[27]. Importantly, activation of NK cells and sensitization of tumor cells to NK cell-mediated killing can contribute to improved tumor cell death [10], [11], [28]. NK cell functions are regulated by a balance of activating and inhibiting signals [29]. Cancer cell resistance mechanisms against NK cell cytotoxicity have been described for several tumors [10], [11]. In this respect, creating an NK cell stimulatory environment through induction of activating NK cell receptors or of their respective ligands expressed by tumor cells [28], [30]–[33], reducing inhibitory signalling [34], [35], or stimulating antibody-dependent cell-mediated cytotoxicity [36], [37] is currently under intense investigation.

Here, we developed an *in vitro* model to dissect the effect of dsRNA-modified acute myeloid leukemic (AML) tumor cell lines upon interaction with primary human NK cells and autologous monocyte-derived immature DC. While current standard therapies for AML can successfully induce remission, the disease is characterized by a high probability of relapse and refractory disease that shorten the survival of AML patients. Evidence of both innate and adaptive immune surveillance and the existence of immune escape mechanisms of the low immunogenic AML cells warrants the application of immune-based strategies to control persisting AML cells [38], [39]. An important observation in patients with AML is the impairment of functional NK cell responses due to a primary NK cell dysfunction, both in the cytotoxic and cytokine producing compartments, and due to the development of NK cell resistance mechanisms by AML cells [32], [40]–[46]. ASI for AML aiming at improvement of recognition by both innate and adaptive immunity and overcoming immune resistance mechanisms is therefore recommended.

Many studies have highlighted the potential role of the synthetic dsRNA analogue polyriboinosinic polyribocytidylic acid (poly(I:C)), as a critical cancer vaccine component as it was shown to exert several anti-tumor functions through its adjuvant effects. Poly(I:C) is an agonist of the innate endosomal TLR3 and the cytosolic melanoma differentiation-antigen-5 (mda-5) receptors [47]. It is a well-described danger signal able to trigger cell death and induce the production of proinflammatory cytokines, including type I interferons (IFN), in various cell types [47]–[53]. Under defined conditions, poly(I:C) is a powerful NK cell activation signal [47], [52], [54]–[60] and it has been shown to efficiently activate and mature DC [61]–[66] and stimulate DC-NK cell interactions [55], [67], [68]. The synthetic dsRNA was also demonstrated to directly [16], [69]–[71] and indirectly [72]–[75] promote cross-presentation of antigens by DC, however, a recent study reported an opposite inhibitory effect on cross-presentation [76]. Of note, different mouse *in vivo* studies demonstrated that tumor cell-associated poly(I:C) induced strong anti-tumor activity. Treatment with dsRNA-associated tumor cells inhibited tumor growth, increased the number of tumor-infiltrating lymphocytes and enhanced the survival of tumor-bearing mice [72], [77].

We have previously reported that AML cells in response to electroporation with poly(I:C) show improved tumor cell immunogenicity, demonstrated by an enhanced capacity to activate and mature DC [53] and induce the release of NK cell IFN-γ [78]. Ensuing from these data, in this study, we examined the effect of poly(I:C)-electroporated AML cells on the phagocytic capacity of immature DC and on the cytotoxic function of NK cells. Using single-cell analysis, we assessed these functionalities in two- and three-party cocultures and checked for type I and II IFN secretion. We demonstrate *in vitro*, an enhanced sensitivity to NK cell-mediated killing and an improved uptake by DC of poly(I:C)-electroporated tumor cells.

## Materials and Methods

### Purification of human NK cells and generation of immature DC

Peripheral blood mononuclear cells (PBMC) were isolated by Ficoll-Paque Plus gradient separation (Amersham Biosciences, Uppsala, Sweden) from fresh heparinised (BD, Plymouth, UK) blood or buffy coat preparations (kindly provided by the Antwerp Blood Transfusion Centre) from healthy donors. Ethics approval for the study was obtained from the Ethics Committee of the Antwerp University Hospital. For experiments with three-party cocultures (DC + NK cells + tumor cells) two consecutive blood draws (day 0 and day 5) per healthy donor were taken, for purification of CD14^+^ monocytes and NK cells, respectively. Two-party cocultures (DC + tumor cells or NK cells + tumor cells) were performed with cell material generated from buffy coat preparations. Purified resting CD56^+^CD3^−^ NK cells were obtained from the PBMC or CD14-negative peripheral blood lymphocyte fraction by using the human negative selection NK cell isolation kit (Miltenyi Biotec, Utrecht, The Netherlands) according to the manufacturer's instructions. NK cells were analysed on a Partec CyFlow ML cytometer (Partec, Münster, Germany), using FITC-, PE- or APC-labeled monoclonal antibodies for CD56 and CD3 (BD Biosciences, Erembodegem, Belgium). Routinely, a purity of 91.2±5.1 % (mean ± SD %, n = 8) viable CD56^+^CD3^−^ NK cells was obtained, without contamination with CD3^+^ T cells. Monocytes were purified from PBMC by immunomagnetic cell selection with CD14 microbeads (Miltenyi Biotec). Subsequently, CD14 positively selected cells (mean purity of 95.8±2.3 %, n = 14) were used for the *in vitro* generation of immature DC. Monocytes were resuspended in RPMI 1640 culture medium (BioWhittaker, Verviers, Belgium) supplemented with 2.5% heat-inactivated human AB (hAB) serum (Sigma-Aldrich, Bornem, Belgium) and seeded in 6-well culture plates (Corning Life Sciences, Schiphol-Rijk, The Netherlands) at a final concentration of 1-1.2×10^6^ cells/mL for 5 days with 800 IU/mL (60 ng/mL) GM-CSF (Gentaur, Brussels, Belgium) and 20 ng/mL IL-4 (Immunotools, Friesoythe, Germany).

### Human cell lines

The human NK-sensitive K562 (chronic myelogenous leukemia in blast crisis) and U-937 (acute myeloid monocytic leukemia) cell lines were used as target cells. Both cell lines were obtained from the American Type Culture Collection (Rockville, MD, USA) and cultured in complete medium consisting of Iscove's modified Dulbecco's medium (IMDM; Lonza, Verviers, Belgium) with L-glutamine (584 mg/L) and 4-(2-hydroxy-ethyl)-1-piperazineethanesulfonic acid (HEPES; 25 mM) supplemented with gentamicin (10 mg/L; Invitrogen, Merelbeke, Belgium), amphotericin B (1 mg/L Fungizone; Invitrogen) and 10% fetal bovine serum (FBS; Perbio Science, Erembodegem, Belgium). The human NK cell line NK92 (Deutsche Sammlung von Mikroorganismen und Zellkulturen, Braunschweig, Germany) for use as positive control in cytotoxicity assays was cultured in MEM alpha medium + GlutaMAX-I (Invitrogen) supplemented with gentamicin and amphotericin B, 12.5% FBS, 12.5% donor equine serum (horse serum, HS; HyClone, Utah, USA) and 100–200 U/mL IL-2 (Invitrogen). All cell lines were maintained in logarithmic phase growth at 37°C in a humidified atmosphere supplemented with 5% CO_2_.

### Fluorescent labeling of tumor cells and DC prior to coculture

In order to simultaneously acquire NK cell cytotoxicity and DC phagocytosis of tumor cells by flow cytometry, NK cells were kept unlabeled, tumor cells were labeled with the green-fluorescent membrane dye PKH67 (Sigma-Aldrich) and DC were stained with cytoplasmic violet-fluorescent CellTracker Violet BMQC dye (Invitrogen). Labeling of tumor cells with PKH67 was carried out according to the manufacturer's instructions and performed prior to electroporation without loss of fluorescence. Briefly, after one wash in serum-free medium, 10×10^6^ tumor cells per mL Diluent C (provided in kit) were rapidly and homogeneously mixed with 2 µM PKH67 dye at room temperature for 5 min. The staining reaction was stopped with FBS (100 %) for 1 min and diluted with an equal volume of IMDM medium with 10% FBS. PKH67-labeled cells were washed three times and checked for fluorescence intensity and viability using propidium iodide (PI; Sigma-Aldrich). Labeled tumor cells were electroporated the same day or were kept in culture at 0.5×10^6^ cells/mL up to the next day for electroporation, without loss of fluorescence and viability. A novel protocol for labeling of DC with CellTracker Violet BMQC was developed by us for use in flow cytometry. The violet dye was brought to a stock concentration of 10 mM with dimethyl sulphoxide (DMSO; Sigma-Aldrich) and diluted with serum-free RPMI medium to a working solution of 5 µM. DC were labeled at 1×10^6^ cells per mL pre-heated working solution at 37°C. After 30 min incubation, cells were washed and resuspended in RPMI medium with 2.5% hAB serum for an additional 30 min incubation at 37°C. Finally, cells were washed twice and checked for viability and labeling efficiency.

### Electroporation of AML cell lines with poly(I:C)

Electroporation of poly(I:C) (Invivogen, Paris, France) was performed as described previously [78]. PKH67-labeled tumor cells were washed twice in serum-free IMDM culture medium and Opti-MEM I medium (Invitrogen) and were resuspended in Opti-MEM I. Subsequently, 0.2 mL of cell suspension was transferred to a 0.4 cm cuvette (Cell Projects, Harrietsham, UK) and mixed with 20 µg poly(I:C). Electroporation was performed using a GenePulser Xcell device (BioRad, Nazareth, Belgium) at predefined settings (300 V, 7 ms). Immediately after electroporation cells were transferred to serum-containing medium and washed twice to remove excess of poly(I:C). Finally, tumor cells were resuspended in IMDM supplemented with 10% FBS or RPMI medium with 2.5% hAB serum, dependent on the experimental setup. Electroporated tumor cells were allowed to rest for 2-4 hours prior to coculture.

### *In vitro* coculturesz

Freshly purified resting NK cells and/or autologous immature DC were put in coculture with mock- (without poly(I:C)) or poly(I:C)-electroporated tumor cells at an effector:target (E:T) cell ratio of 1:1 for two-party and 1∶1∶1 for three-party cocultures (0.2×10^6^ cells per cell type) in a final volume of 600 µL in sterile polypropylene tubes (Greiner bio-one, Menen, Belgium). When DC were added, cocultures were always held in RPMI medium supplemented with 2.5% hAB. In absence of DC, cells were cultured in RPMI with 2.5% hAB or IMDM medium with 10% FBS. After overnight coculture (mean duration of 16.4±1.6 h, n = 12) at 37°C, tubes were centrifuged and supernatant was collected and stored at −20°C for future analysis. Cell pellets were immediately used for flow cytometric detection of cytotoxicity and phagocytosis.

### Flow cytometric detection of NK cell-mediated cytotoxicity and phagocytosis

**Figure 1:**
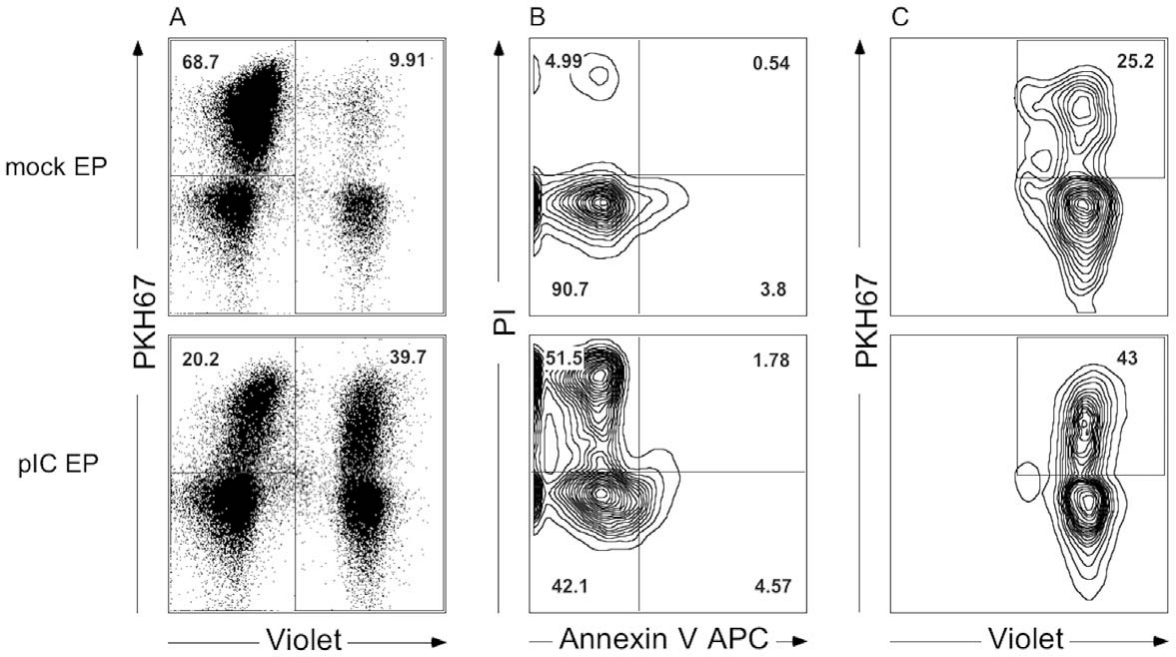
Simultaneous flow cytometric detection of NK cell cytotoxicity and DC phagocytosis of tumor cells. Plots show the flow cytometric analysis of three-party cocultures of NK cells + DC with mock-electroporated tumor cells (upper panel) and with poly(I:C)-electroporated tumor cells (lower panel). (**A**) Target cells labeled with PKH67 dye (upper left gate), DC labeled with CellTracker Violet BMQC dye (right gate) and unlabeled NK cells (lower left corner) can be explicitly discriminated. After exclusion of doublets (FSC-area versus FSC-width), (**B**) viability (annexin V^−^ PI^−^, lower left quadrant) of PKH67^+^ tumor cells (A, upper left gate) can be determined and used for calculating the % killing. (**C**) Phagocytosis of PKH67^+^ tumor cells by violet-labeled DC is expressed as the % PKH67^+^violet^+^ cells within the violet^+^ DC population (A, right gate), selected for single cells. Abbreviations: mock EP, electroporated without poly(I:C); pIC EP, electroporated with poly(I:C).

Overnight cocultures were stained for cell death with annexin V (APC-conjugated; BD Biosiences) in annexin V binding buffer (BD Biosciences) and PI. Acquisition was performed on a Partec CyFlow ML (Partec) or a FACSAria II (BD Biosciences) multicolor cytometer. Usually, 20 000 PKH67^+^ cells per sample were acquired. Flow cytometric data analysis was performed using FlowJo version 8.8.6 (Treestar, Ashland, USA). Figure 1 shows an example of gating strategy for determination of NK cell cytotoxicity and DC phagocytosis of tumor cells. The first gate was always put on single cells based on the forward scatter (FSC)-Area versus FSC-Width scatter profile. Cytotoxicity was determined based on the viability (annexin V^-^ PI^-^) of PKH67^+^ target cells. The percentage killing was calculated taking into account the viability of mock- or poly(I:C)-electroporated tumor cells after culture with NK cells and/or DC, relative to respectively mock- or poly(I:C)-electroporated tumor cells cultured alone. Results are depicted using the following equation: % killing  = 100−[(% annexin V^-^ PI^-^ tumor cells with effector cells/% annexin V^-^ PI^-^ tumor cells without effector cells)×100]. For each experiment, the human NK cell line NK92 was stimulated with mock-electroporated tumor cells and IL-2 (Invitrogen) as a positive control of the killing assay. Phagocytosis of PKH67^+^ tumor cells by violet-labeled DC was expressed as the % PKH67^+^violet^+^ cells within the violet+ DC population. Parallel to cocultures at 37°C, negative controls at 4°C were carried out.

### Cytokine secretion profile

Secretion of IFN-α and IFN-γ in supernatant of overnight cocultures was determined using a multiplex FlowCytomix kit (Bender MedSystems, Vienna, Austria) according to the manufacturer's instructions. Flow cytometric detection of cytokines was assessed at once in thawed non-diluted supernatant. Standard curves of each analyte reached a top concentration of 20 000 pg/mL. All samples were acquired on a FACScan cytometer (BD Biosciences, San Jose, CA, USA) and data were analysed using FlowCytomix Pro 2.3 software (Bender MedSystems).

### Statistical analysis

GraphPad Prism 5.0 software (GraphPad Software, San Diego, USA) was used for graphical data representations and statistical computations. Statistical analysis was performed using a paired non-parametric Wilcoxon Signed-Rank test or Spearman rank correlation test, where appropriate. P-value <0.05 was considered statistically significant.

## Results

### dsRNA transfection of AML cells enhances their susceptibility to NK cell-mediated killing

Previously, we showed that AML cells in response to poly(I:C) loading by electroporation become apoptotic and secrete IFN-α [53]. Through secretion of type I IFN, they are able to trigger substantial NK cell IFN-γ release without the need of additional triggering signals [78]. In this study, we investigated the effect of these dsRNA-modified AML cells on the cytotoxic function of NK cells and hypothesized that poly(I:C)-induced apoptosis would increase the sensitivity to NK cell-mediated killing. Poly(I:C)-induced apoptosis was determined for each electroporation. Table 1 depicts the viability, defined as the percentage annexin V-negative and PI-negative cells, of K562 and U-937 cells upon mock and poly(I:C) electroporation after overnight culture with or without effector cells. Mock-electroporated K562 (n = 10) and U-937 (n = 12) cell lines remained viable for 82.1±7.3% and 88.4±8.9% (mean ± SD), respectively. Viability of poly(I:C)-electroporated K562 cells was lowered to 59.2±21.3%, whereas U-937 cells became even more apoptotic with only 31.8±11.4% remaining viable.

**Table 1:** Viability (% annexin V^−^ PI^−^) of mock- and poly(I:C)-electroporated tumor cells after overnight culture with and without effector cells.

Effectors	K562		U-937	
	mock EP	pIC EP	mock EP	pIC EP
-	82.1±7.3	59.2±21.3	88.4±8.9	31.8±11.4
NK	74.2±10.5	42.0±19.8	77.6±14.4	20.4±11.6
NK+DC	76.5±9.8	37.4±19.2	71.5±18.2	20.3±12.9

Results are expressed as a mean value (n between 7 and 12) ± SD of the percentage annexin V^−^ PI^−^ PKH67^+^ single cells after overnight culture at a ratio of 1∶1 for NK + tumor cells and 1∶1∶1 for NK + DC + tumor cells.

**Figure 2:**
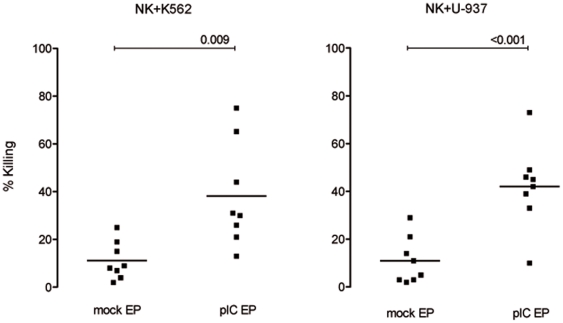
Poly(I:C)-electroporated tumor cells are more susceptible to NK cell-mediated killing compared to mock-electroporated tumor cells. Percentage killing of mock- and poly(I:C)-electroporated tumor cell lines K562 and U-937 by purified NK cells based on the viability (annexin V^−^ PI^−^) of PKH67^+^ target cells. Cocultures were kept overnight at a 1∶1 NK:tumor cell ratio. Horizontal lines represent mean of 8 donors. Differences are statistically significant if p<0.05. Abbreviations: mock EP, electroporated without poly(I:C); pIC EP, electroporated with poly(I:C).

cytokinesPresented in figure 2, overnight coculture of purified NK cells with mock-electroporated K562 or U-937 cells at an E:T ratio of 1∶1 resulted in respectively 11±8% (mean ± SD; n = 8) and 11±10% (n = 8) killing. Non-electroporated tumor cells were killed by NK cells at a similar rate as to that of mock-electroporated tumor cells for the two cell lines (data not shown). Importantly, killing of poly(I:C)-electroporated target cells was significantly higher compared to mock-electroporated target cells (p = 0.009 for K562 and p<0.001 for U-937). Poly(I:C)-electroporated K562 and U-937 cells were killed for 38±22% and 42±18% respectively. For each independent experiment, the cytotoxic NK cell line NK92 was cocultured with mock-electroporated target cells at an E:T ratio of 1∶1 in the presence of IL-2 serving as a positive control. NK92 effector cells killed K562 cells for 56±23% (n = 8) and U-937 cells for 70±9% (n = 9), with a residual viability of 36.2±18.1% and 27.1±10.5%, respectively.

### Poly(I:C) electroporation of AML cells improves subsequent phagocytosis by DC

Poly(I:C)-electroporated AML cells are capable of stimulating immature DC as evidenced by an increased expression of major histocompatibility complex (MHC) and costimulatory molecules, production of proinflammatory cytokines and an increase of Th1-polarizing capacity [53]. Here, we examined if poly(I:C) electroporation of AML cells has an influence on their subsequent phagocytosis by immature DC.

**Figure 3:**
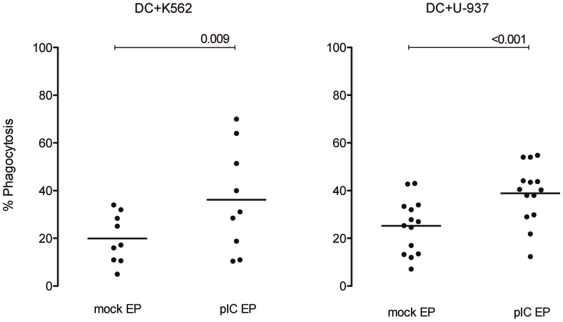
Phagocytosis of poly(I:C)-electroporated tumor cells by DC is higher compared to mock-electroporated tumor cells. Percentage phagocytosis of mock- and poly(I:C)-electroporated tumor cell lines K562 (n = 9) and U-937 (n = 14) by immature DC is shown. Phagocytosis is expressed as the percentage PKH67^+^ DC within the violet^+^ DC after overnight coculture at a 1∶1 DC:tumor cell ratio. Horizontal lines represent means and differences are statistically significant if p<0.05. Abbreviations: mock EP, electroporated without poly(I:C); pIC EP, electroporated with poly(I:C).

As depicted in figure 3, flow cytometric analysis revealed that 19.9±10.3% K562 mock-electroporated (mean ± SD; n = 9) and 25.2±11.4% U-937 mock-electroporated (n = 14) tumor cells were phagocytosed by DC after overnight coculture at a 1∶1 DC:tumor cell ratio. Uptake of non-electroporated tumor cells was similar to that of mock-electroporated tumor cells, for the two cell lines (data not shown). Interestingly, poly(I:C)-electroporated tumor cells were phagocytosed more efficiently than mock-electroporated tumor cells. A mean of 36.1±21.9% poly(I:C)-electroporated K562 cells (range 10–70%, p = 0.009) and 38.9±12.3% poly(I:C)-electroporated U-937 cells (range 12–55%, p<0.001) was taken up by DC.

### Tumor cell killing but not phagocytosis by DC is improved in three-party cocultures

**Figure 4:**
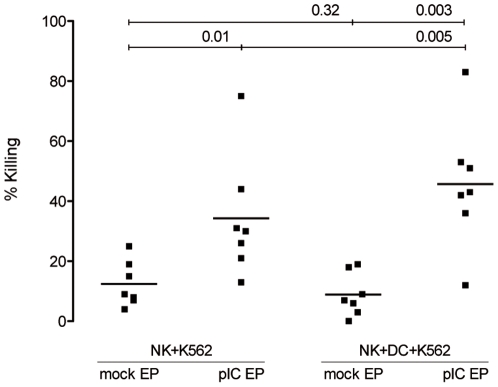
Improved killing of K562 cells by NK cells in the presence of DC when K562 cells are sensitized with poly(I:C). Percentage killing of mock- and poly(I:C)-electroporated K562 cells by NK cells or by NK cells and DC. Cocultures were kept overnight at a 1∶1 NK:K562 or a 1∶1∶1 NK:DC:K562 cell ratio. Horizontal lines represent mean of 7 donors. Differences are statistically significant if p<0.05. Abbreviations: mock EP, electroporated without poly(I:C); pIC EP, electroporated with poly(I:C).

cytokinesTo examine functional cross-talk between NK cells and DC, we simultaneously examined the killing and phagocytosis of AML cell lines in coculture with NK cells and autologous immature DC. Interestingly, the presence of DC in NK cell:K562 cell cocultures significantly improved tumor cell killing, provided that K562 cells were upfront electroporated with poly(I:C) (figure 4; p = 0.32 for mock-electroporated and p = 0.005 for poly(I:C)-electroporated K562 cells). This effect was not significant for U-937 (p = 0.15 for mock-electroporated and p = 0.46 for poly(I:C)-electroporated U-937 cells; data not shown). Next, we hypothesized that improved killing of poly(I:C)-electroporated tumor cells by NK cells could lead to an even higher uptake of apoptotic cells by DC. However, we could not see any improvement of phagocytosis of mock- nor poly(I:C)-electroporated tumor cells if NK cells were added to the coculture (p = 0.17 and p = 0.26 for K562 and p = 0.68 and p = 0.09 for U-937, respectively; data not shown).

### U-937 cells secrete IFN-α upon poly(I:C) electroporation and induce secretion of NK cell IFN-γ

**Figure 5:**
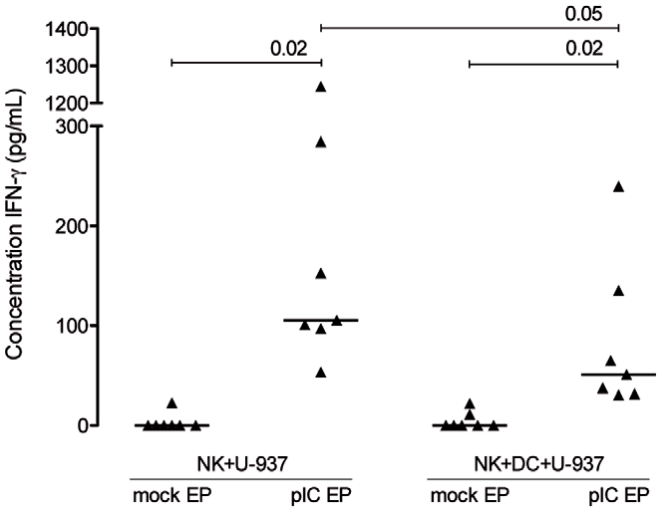
Release of NK cell IFN-γ upon stimulation with poly(I:C)-electroporated U-937 cells. Concentration of IFN-γ secreted by NK cells in two- and three-party cell cultures. Cocultures were held overnight at a 1∶1 NK:U-937 or a 1∶1∶1 NK:DC:U-937 cell ratio. Horizontal lines represent median values for 7 donors. Differences are statistically significant if p<0.05. Abbreviations: mock EP, electroporated without poly(I:C); pIC EP, electroporated with poly(I:C).

Concentrations of IFN-α and IFN-γ were determined in non-diluted supernatant of overnight cocultures. Concomitant to our previous data [53], [78], poly(I:C) electroporation of U-937 cells but not K562 cells, induced high levels of IFN-α secretion (1490.99±633.53 pg/mL, mean ± SD, n = 7; p = 0.02). The concentration of IFN-α did not change when poly(I:C)-electroporated U-937 cells were cocultured with NK cells and/or DC. As anticipated from our previous results [78], stimulation of NK cells with IFN-α-secreting poly(I:C)-electroporated U-937 cells, in contrast to K562 cells (that do not secrete IFN-α upon poly(I:C) electroporation), led to a significant IFN-γ secretion (p = 0.02; figure 5). In three-party cocultures consisting of K562 cells, also no IFN-γ was detected. Addition of DC to NK cell:U-937 poly(I:C)-electroporated cocultures negatively influenced the IFN-γ concentration (p = 0.05; figure 5), however it remained significantly higher compared to mock-electroporated conditions (p = 0.02; figure 5).

### Relations between cytotoxicity, phagocytosis and cytokine secretion

**Figure 6:**
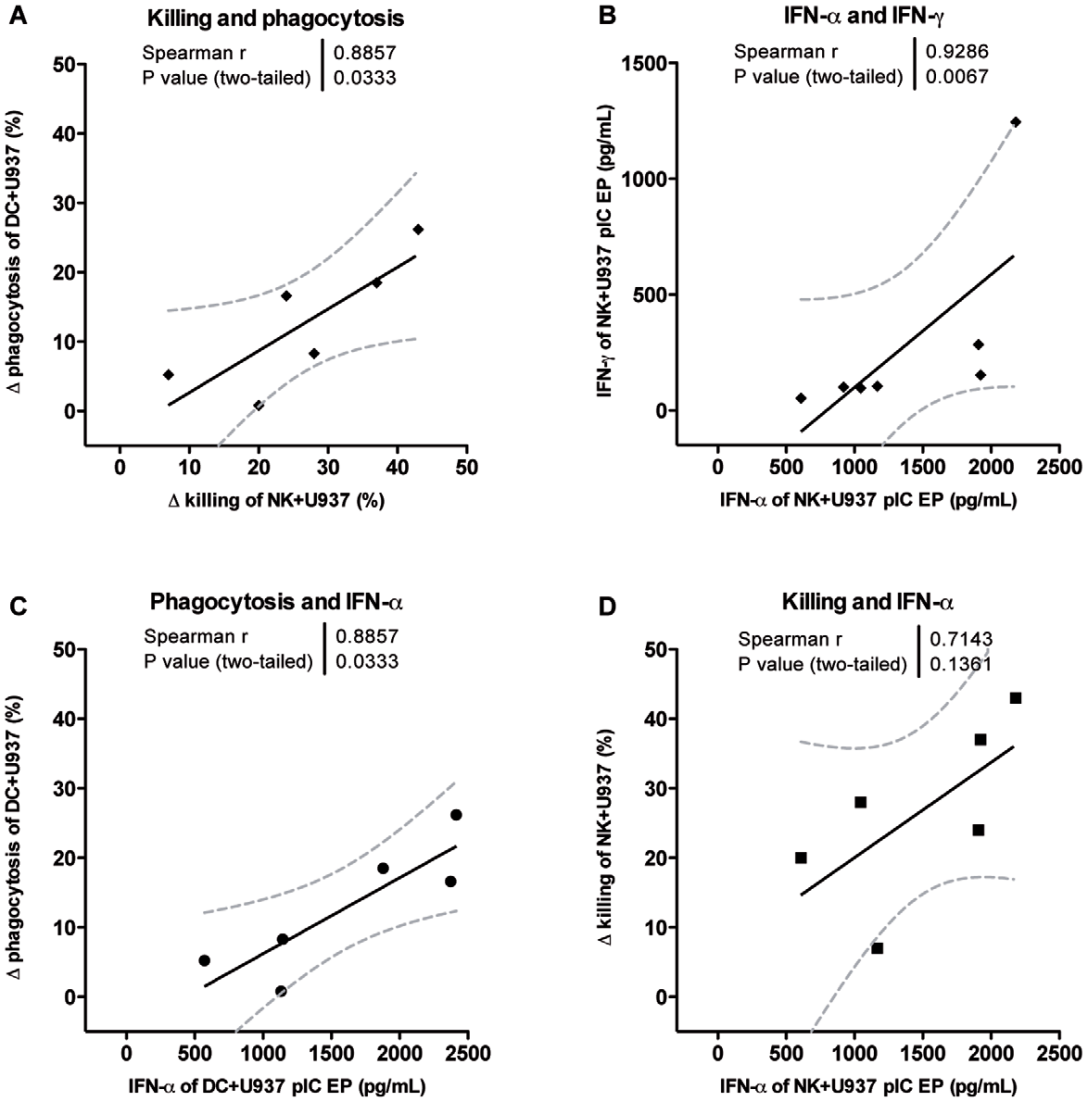
Correlations of functional properties of NK cells and DC regarding poly(I:C) electroporation of the U-937 cell line. (**A**) Positive correlation between the effect of poly(I:C) electroporation of U-937 cells on the NK cell killing capacity and the DC phagocytic function, expressed as the difference (delta) in functions between mock- and poly(I:C)-electroporated U-937 cells. (**B**) The concentrations IFN-α and IFN-γ in supernatant of NK cell:U-937 poly(I:C)-electroporated cocultures are strongly correlated. The improved (**C**) phagocytosis by immature DC and (**D**) killing by NK cells are in positive relation with the concentration IFN-α secreted by poly(I:C)-electroporated U-937 cells. Spearman rank correlations were calculated for defined functions. Abbreviations: mock EP, electroporated without poly(I:C); pIC EP, electroporated with poly(I:C); Δ killing  =  % killing of poly(I:C)-electroporated tumor cells - % killing of mock-electroporated tumor cells; Δ phagocytosis  =  % phagocytosis of poly(I:C)-electroporated tumor cells - % phagocytosis of mock-electroporated tumor cells.

Both killing by NK cells and phagocytosis by DC are significantly improved when AML cells are electroporated with poly(I:C). Focusing on the effect of poly(I:C) electroporation, we looked at the difference of killing and phagocytosis between mock- and poly(I:C)-electroporated tumor cells, depicted as Δ killing (% killing of poly(I:C)-electroporated tumor cells - % killing of mock-electroporated tumor cells) and Δ phagocytosis (% phagocytosis of poly(I:C)-electroporated tumor cells - % phagocytosis of mock-electroporated tumor cells). Comparing the killing capacity in NK:tumor cell cocultures with the phagocytic capacity in DC:tumor cell cocultures, we observed a significant positive correlation for the U-937 cell line (r = 0.89, p = 0.03; figure 6A), but not for K562 cells (r = −0.67, p = 0.23). Interestingly, we also demonstrate correlations with reference to the effect of poly(I:C) electroporation of the U-937 cell line and the cytokines IFN-α and IFN-γ. A positive correlation was found between the poly(I:C)-induced enhanced killing of U-937 cells by NK cells and the concentration of IFN-α secreted by U-937 cells (r = 0.71, p = 0.14; figure 6D). The IFN-α concentration was also found to be positively correlated with the improved uptake of U-937 cells by DC upon poly(I:C) electroporation (r = 0.89, p = 0.03; figure 6C). With regard to the IFN-γ concentration, detected in cocultures with NK cells and poly(I:C)-electroporated U-937 cells, we demonstrate a positive relationship with the improved killing in NK cell:tumor cell cocultures (r = 0.60, p = 0.24) and with the improved uptake of U-937 cells in NK cell:DC:tumor cell cocultures (r = 0.77, p = 0.10; data not shown). Finally, the concentrations IFN-α and IFN-γ found in cocultures of NK cells and poly(I:C)-electroporated U-937 cells are strongly correlated (r = 0.93, p = 0.007; figure 6B).

## Discussion

We recently showed that human AML cells in response to electroporation with the synthetic dsRNA poly(I:C) enhance their capacity to act on DC and NK cells. AML cells become highly susceptible to apoptosis and subsequently secrete marked amounts of IFN-α [53], [78]. Stimulation of immature DC with poly(I:C)-electroporated AML cells results in maturation of DC as evidenced by an increased expression of MHC and costimulatory molecules, production of proinflammatory cytokines and an increase of Th1-polarizing capacity [53]. Through secretion of IFN-α, these tumor cells are capable of triggering NK cells to secrete substantial amounts of IFN-γ, without the need of additional triggering signals [78]. Together, these data signify that mimicking viral infection of AML tumor cells through introduction of dsRNA in the cytosol by poly(I:C) electroporation, leads to a strong improvement of tumor cell immunogenicity and may provide a local DC- and NK cell-stimulating environment. To further invigorate the potential of these immunogenic tumor cells, we explored the effect of poly(I:C) electroporation of AML cells on the killing capacity of NK cells and the phagocytic potential of DC.

Here, we demonstrate that through electroporation with poly(I:C), AML cell lines become highly susceptible to NK cell-mediated killing and phagocytosis by immature DC. Interestingly, NK cells are capable of killing poly(I:C)-electroporated U-937 cells to the same extent as the classical NK-sensitive K562 cell line, despite the high expression of MHC class I molecules on U-937 cells which is known to counteract NK cell-mediated killing under normal circumstances. Noteworthy, the magnitudes of killing by purified NK cells in this study, at an E:T cell ratio of 1∶1, have been achieved in the past mostly at higher E:T ratio's and/or in the presence of NK cell activating signals (e.g. maintenance concentration of IL-2, polyclonally activated NK cells). The presence of IFN-α secreted by the tumor cells could support the hypothesis of higher NK cell cytotoxicity and better internalization by DC. Type I IFNs can exert both direct effects on tumor cells and indirect effects on host immune cells that play a central role in the overall anti-tumor response [79]. IFN-α is a known modulator of cytotoxicity and has been shown to stimulate cross-presentation of tumor antigens upon internalization by DC [80], [81]. In line with these observations, we could demonstrate a positive correlation between IFN-α secretion following poly(I:C) treatment of U-937 cells and both NK cell-mediated killing and phagocytosis by DC. In addition, the amount of IFN-γ detected in cocultures of NK cells and poly(I:C)-electroporated U-937 cells strongly correlated with the concentration of IFN-α, which may be the result of a direct immunostimulatory action of IFN-α on the IFN-γ secretory capacity of NK cells. Finally, we also observed a strong correlation between the enhanced NK cell-mediated cytotoxicity and the improved uptake of U-937 cells by DC. In contrast to U-937 cells, K562 cells are unable to secrete IFN-α upon poly(I:C) electroporation and subsequently are powerless to trigger IFN-γ release from NK cells. Nevertheless, we found that poly(I:C)-electroporated K562 cells are still more susceptible to NK cell cytotoxicity and to uptake by DC as compared to their untransfected counterparts. This finding argues against a direct role for IFN-α in potentiating NK cell-mediated killing of tumor cells following poly(I:C) electroporation. This is in line with other studies [82]-[84] demonstrating an IFN-α independent improvement of NK cell-mediated lysis of specific tumor cells. Based on previous studies from our laboratory, similar conclusions can be anticipated for the role of IFN-α in enhancing tumor cell uptake by DC [53] and in stimulating IFN-γ release from NK cells [78]. Neither improvement of phagocytosis, nor IFN-γ secretion by NK cells was observed following poly(I:C) electroporation of the promyelocytic leukemic cell line NB-4, despite the potent induction of IFN-α. These data indicate that the pro-immunogenic effects of poly(I:C) electroporation can evolve independent of IFN-α.

Taken together, our results provide further evidence that poly(I:C) could find application as a potent immunostimulatory adjuvant that may help overcoming resistance to tumor cell killing by improving NK cell-mediated cytotoxicity. In addition, we show that poly(I:C) treatment of tumor cells results in improved phagocytosis of tumor cells by DC, which may ultimately lead to improved tumor antigen presentation by DC and stimulation of specific T cells. Further research is needed to unravel the mechanisms used by DC and NK cells involved in these phenomena. Paths to explore include the expression upon poly(I:C) electroporation of several ‘eat me’ signals (e.g. phosphatidylserine, calreticulin, heat-shock proteins, high-mobility group box-A protein), that are emitted during immunogenic cell death to facilitate their recognition and phagocytosis by neighbouring immune cells [1], [5], [13]. Analysis of the expression of the activating receptor NKG2D on NK cells and its ligands (MHC class I chain-related (MIC) A/B and UL16 binding proteins (ULBPs)), which are down-regulated or absent on AML cells [32], [44]-[46], could also provide a possible explanation for the improved sensitivity towards NK cells. Finally, mapping the overall cytokine profile of dsRNA-modified tumor cells cocultured with DC and NK cells would be of essential value to further dissect the mechanisms of action.

Arising from the observation that poly(I:C)-loaded tumor cells are both killed and phagocytosed more efficiently, the potential bidirectional cross-talk between NK cells and autologous DC was investigated in three-party cocultures. We hypothesized that improved tumor cell killing by NK cells and/or the release of both IFN-α and IFN-γ (two important phagocyte-stimulating factors), could provide immature DC with even more tumor cell material to be internalized. However, the presence of NK cells (for both K562 and U-937) and/or IFN-α and IFN-γ (for U-937 only), did not further improve the phagocytosis of poly(I:C)-electroporated tumor cells. Vice versa, several studies have shown that DC are capable of augmenting the cytotoxic function of NK cells, putting forward an important pathway of stimulating innate anti-tumor effects. DC-mediated NK cell activation was shown to be cell contact dependent and to rely on defined stimuli [2]. Basically, cytolytic activity was augmented if NK cells were cultured with DC after exposure to various activation/maturation signals such as TLR-dependent microbial stimuli [85]–[88], virally infected tumor cells [89], [90] or immune stimulating cytokine cocktails [86], [91]–[93]. In line with these observations, we showed that monocyte-derived immature DC further enhanced the killing of K562 cells, under the condition that these tumor cells were upfront electroporated with poly(I:C). This effect occurred independent of IFN-α, since no IFN-α was detected in the supernatants of these cocultures, as described earlier. Based on previous reports about NK cell-mediated killing of immature DC [42], [94]–[99], we also checked for DC viability in the different cocultures. After overnight culture with tumor cells and/or NK cells, we could not detect any differences in DC viability compared to unstimulated DC (not shown), ruling out killing of DC.

In addition to potentiating the NK cell cytolytic function, it is described that DC may trigger the release of IFN-γ from NK cells [65], [88]–[90], [100]–[103]. In this study, no IFN-γ was detected in the supernatants of three-party cocultures containing the K562 cell line. Moreover, high IFN-γ concentration in conditions with IFN-α-secreting poly(I:C)-electroporated U-937 cells, was significantly lowered when DC were added to the coculture. In a study by Bontkes et al [100], both the IFN-γ production as well as the cytolytic response towards K562 cells was significantly increased after stimulation with IL-12- and/or IL-18-secreting DC (through electroporation with IL-12 and IL-18 mRNA). We did not detect IL-12 and IL-18 in these culture supernatants (not shown), which would be in support of the absence of IFN-γ in these three-party conditions. This suggests that additional mechanisms may be responsible for the improved killing of poly(I:C)-electroporated K562 cells in the presence of DC, but the exact pathways underlying this interaction remain to be uncovered.

Regarding the scope of reaching multiple immune cells to improve anti-tumor efficacy, the activation of DC and NK cells is an attractive modality for cancer immunotherapy. The key to effective ASI relies on the creation of optimal recognition and ensuing communication between the tumor and immune cells in order to obtain a sustained anti-tumor response. In summary, our recent findings show that mimicking viral infection of AML cells through transfection of dsRNA by electroporation with poly(I:C) improves their recognition by both DC and NK cells. Transfected tumor cells are able to activate DC and NK cell functions and stimulate NK-DC cross-talk in terms of tumor cell killing. This dual action on DC and NK cell immunity may provide a way to overcome immune evasion mechanisms by leukemic tumor cells. These data strongly support the use of poly(I:C) as a cancer vaccine component and provide a framework for the generation of anti-tumor immunity against low/non immunogenic tumor cells. Understanding the mechanisms that cause these observations will provide insight into the future optimization and development of novel vaccination regimens.
